# The role of vitamin D testing and replacement in fibromyalgia: a systematic literature review

**DOI:** 10.1186/s41927-018-0035-6

**Published:** 2018-10-05

**Authors:** Shawn D. Ellis, Sam T. Kelly, Jonathan H. Shurlock, Alastair L. N. Hepburn

**Affiliations:** 1 Department of Oncology, Royal Berkshire Hospital, Reading, RG1 5AN UK; 2 Department of Medicine, Royal Sussex County Hospital, Brighton, BN2 5BE UK; 3 Department of Rheumatology, Worthing Hospital, Worthing, BN11 2DH UK

**Keywords:** Fibromyalgia, FMS, Vitamin D, Hypovitaminosis D, Systematic review

## Abstract

**Background:**

Fibromyalgia is a debilitating condition, characterized by extensive muscular pain and fatigue. Vitamin D is essential for overall health, with ubiquitous involvement in various inflammatory and pain pathways. Little is known about its role in fibromyalgia. We performed a systematic literature review to determine if vitamin D contributes to the pathology and disability of patients with fibromyalgia, and to assess the role of vitamin D supplementation in disease management.

**Methods:**

We searched Medline, EMBASE and the Cochrane Library for clinical studies and randomized controlled trials published in English during January 2000 to June 2017, using the terms vitamin D or hypovitaminosis D combined with fibromyalgia or FMS. References were reviewed manually and articles were only included if they were specific in their diagnosis of fibromyalgia and used appropriate control groups.

**Results:**

Four hundred and sixty-six studies were retrieved, of which fourteen fulfilled the inclusion criteria. Six studies, of which two had the best quality evidence, found that patients with fibromyalgia have low levels of vitamin D compared to healthy controls. Conflicting results were obtained on the effect of vitamin D on pain or symptom control, with no clear consensus as to the role of supplementation in the management of fibromyalgia.

**Conclusions:**

Our results highlight an association between vitamin D deficiency and fibromyalgia. However, its role in the pathophysiology of fibromyalgia and the clinical relevance of identifying and treating this requires further elucidation with appropriately controlled studies.

## Background

Fibromyalgia syndrome (FMS) is a common disorder, affecting 2–3% of the population, that is characterized by chronic widespread muscular pain, generalized weakness and occasional bone pain [1]. The American College of Rheumatology (ACR) devised the 1990 criteria for FMS diagnosis based on the aforementioned symptoms being present for three months or more [1], and was updated in 2010 to include the exclusion of other disorders that might otherwise mimic FMS [2]. Interestingly, these symptoms are also found in individuals with low levels of vitamin D, particularly fatigue and widespread muscle pain and weakness [3].

Vitamin D is a pleiotropic hormone with a critical role in modulating several inflammatory and pain pathways in addition to calcium homeostasis. Observational studies suggest an association between vitamin D deficiency and chronic pain, most promisingly in fibromyalgia [4]. Indeed, it has been hypothesized that vitamin D has anti-inflammatory properties that contribute to relieving pain. In vitro studies have found that the vitamin can reduce prostaglandin E2 (PGE2) synthesis to down-regulate proinflammatory pathways [5] and its supplementation can improve musculoskeletal pain [6]. The anti-inflammatory effects of vitamin D have also been attributed to its impact on T cell differentiation and the development of regulatory T cell populations that modulate pro-inflammatory Th1 and Th17 cells [7–9].

Testing for serum vitamin D levels has increased significantly in recent years [10, 11], especially in patients with musculoskeletal pain syndromes [3, 12] and those with other medically unexplained symptoms [13], presumably in the search for a potentially reversible cause. In parallel, there has been a rise in interest in this area by the pharmaceutical industry, with a corresponding increase in the number of licensed vitamin D preparations, as well as ‘over the counter’ supplements [14]. Taken together, these factors have significant health economic implications.

This review aims to identify and appraise the available evidence comparing vitamin D levels in FMS patients with healthy controls, and to evaluate the efficacy of supplementation in deficient FMS patients. Thus, it aims to address whether FMS patients will benefit from vitamin D deficiency testing and treatment.

## Methods

This review followed the Preferred Reporting Items for Systematic Review and Meta-Analyses (PRISMA) guidelines, employing the PRISMA-TC 2015 checklist [15, 16].

### Eligibility criteria

Included in the review were observational studies that prospectively compared blood serum levels of vitamin D (measured by 25(OH)D) in FMS patients with age and gender-matched healthy controls, and also randomized control trials (RCTs) that measured the correlation of vitamin D levels with changes in symptom severity in vitamin D deficient FMS patients after administration of supplementation compared with placebo. Additional inclusion criteria for these two types of studies were limited to being published in the English language, investigating human subjects of 18 years or more, diagnosis of chronic pain specific to FMS and being published between the time period of January 2000 to June 2017. Studies were excluded from the review if they had an ambiguous definition of FMS, were published before the aforementioned dates or were published in a non-English language.

### Search strategy

Three independent reviewers (SE, SK and JS) performed a database search across Medline, EMBASE and the Cochrane Library, using the following terms: “vitamin D” or “hypovitaminosis D” combined with “fibromyalgia” or “FMS.” Titles of retrieved studies were screened, after which abstracts and full texts of remaining studies were cross-examined according to the review inclusion criteria. A manual search of all included bibliographies was carried out to identify any omitted articles.

### Quality assessment

Included studies were assessed using an adapted version of the Newcastle-Ottawa checklist [17], which is specific for the reporting of cross-sectional observational studies in order to avoid conclusions drawn from low-quality research. This comprised of three distinct areas of quality: (1) selection of the groups involved (score of: 0–4), (2) quality of the adjustment for confounding variables (score of: 0–2), and (3) ascertainment of the outcome measure of interest for the groups (score of: 0–3) thus producing a cumulative quality score for which the maximum is 9 and reflects the greatest possible methodological research quality. Similarly, RCTs were assessed against the Critical Appraisal Skills Program (CASP) which evaluates the rationale for the research, the effective randomization and blinding techniques employed, assessment of statistical techniques used, evaluation of the practical application of research population to target population who would eventually benefit from the intervention and appraisal of harms and cost-effectiveness.

### Data extraction

The following information was obtained from each study: name of first author, year of publication, country in which the research was conducted, type of study design, sample size and characteristics. Outcome measures extracted included mean or median 25(OH)D or 1,25(OH)D levels, frequency of hypovitaminosis of FMS and control populations and any correlations of vitamin D levels with disease activity scores. RCTs were also searched for initial vitamin D levels, method and regimen of supplementation, post-supplementation vitamin D levels and correlation values with symptom severity measures. Information was also collected regarding the country that the research was conducted in and the gender and ethnicities of the participants.

## Results

### Search strategy

**Fig. 1:**
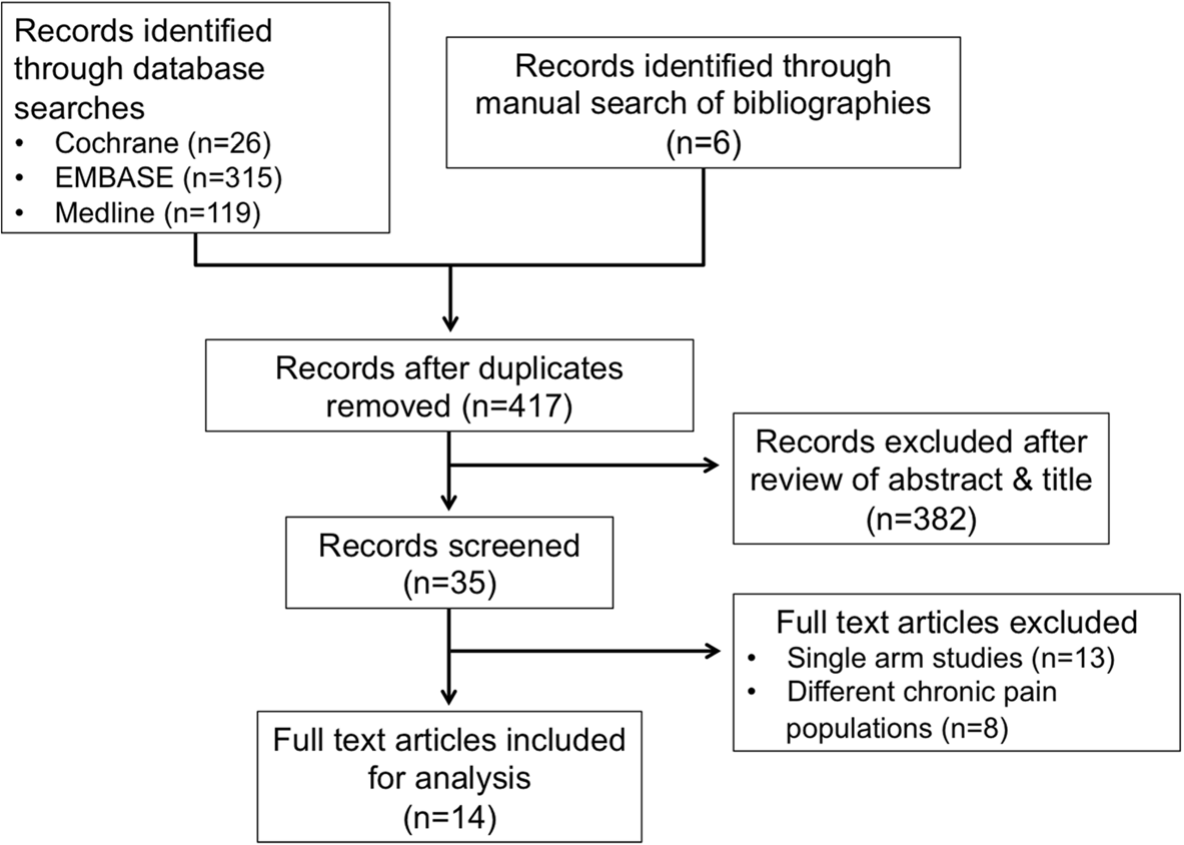
Flow diagram of the systematic literature review’s inclusion and exclusion process

Four hundred and sixty-six studies were retrieved by the database and manual search, 382 of which were excluded due to title or study design. 49 duplicated articles were also removed. The full texts of the remaining 35 studies were read and their content cross-referenced with the inclusion criteria, leaving 14 relevant studies (Fig. 1). Studies were excluded for lack of control groups and non-specific diagnosis of FMS pain.

### Study characteristics

Of the 14 included studies, 12 were cross-sectional [12, 18–27], comparing mean values of vitamin D in diagnosed FMS populations with healthy controls, one was a RCT [28] and one published data from both a cross-sectional study and RCT [29]. An analysis of the included studies is listed in Table 1.

**Table 1:** Summary of Included Studies

Author	Year	Study Design	Country	Definition of FMS	Control Characteristic	Season of Measurement	Age of Patients	Age of Controls	Sex	No. Patients	No. of Controls	Threshold of Vit D deficiency (ng/ml)	Method of Vit D measure	NC-Ottawa Scale
Al-Allaf et al.	2003	CSS	UK	ACR 1990	Age & sex matched healthy controls	No mention	42.5 ± 3.6	42.5 ± 4.3	F	40	37	8	No mention	7
Warner et al.	2008	CSS & RCT	USA	ACR 1990. Tender points exam omitted	Osteoarthritis affecting fewer than 3 joints. Not age or sex matched.	Spring & Summer (May-Aug)	54.4 ± 11.7	66.4 ± 10.5	M + F	184	104	20	Liquid chromatography	5 N/A for RCT component
Tandeter et al.	2009	CSS	Israel	ACR 1990	Age & sex matched healthy controls	No mention	43.83 ± 7.57	40.37 ± 9.85	F	68	82	20	ELISA	6
Atherton et al.	2009	CSS	UK	ACR 1990	Participants without chronic pain in 1958 nationwide biomedical survey	Autumn–Spring (Sep-Mar)	45	45	M + F	743	5593	20	ELISA	7
Ulusoy et al.	2010	CSS	Turkey	ACR 1990	Age & sex matched healthy controls	Spring & Summer (May–June)	20–40	20–40	F	30	30	20	Liquid chromatography	7
McBeth et al.	2010	CSS	Italy, Belgium, Poland, Sweden, UK, Spain, Estonia, Hungary	ACR 1990	Individuals with no pain	No mention	40–79	40–79	M + F	263	1262	20	radioimmunoassay	7
de Rezende Pena et al.	2010	CSS	Brazil	ACR 1990	Individuals with no pain	Summer in S. hemisphere (Nov-Jan)	18–60, 44.87 ± 8.57	18–60, 32.03 ± 10.57	M + F	87	92	15	Liquid chromatography	5
Olama et al.	2013	CSS	Egypt	ACR 1990	Age & sex matched healthy controls	Spring & Summer (May–July)	32.3 ± 9.4	33.1 ± 9.7	F	50	50	20	ELISA	7
Okumus et al.	2013	CSS	Turkey	ACR1990	Mechanical low back pain; age & sex matched	Winter & Spring (Nov-Mar)	41.23 ± 4.8	39.48 ± 4.08	F	40	40	37.5	radioimmunoassay	5
Mateos et al.	2014	CSS	Spain	No mention	Age & sex matched healthy controls	Autumn & Winter (Nov-Dec)	51 ± 9.6	51.3 ± 9.9	F	205	205	8	chemiluminescence	5
Wepner et al.	2014	RCT	Austria	ACR 1990 & 2010	Individuals with FMS not receiving vit D supplement	Summer	48.37 ± 5.301	48.37 ± 5.301	M + F	15	15	32	No mention	N/A
Okyay et al.	2016	CSS	Turkey	ACR1990	Age & sex matched healthy controls	Summer	36.97 ± 8.95	35.75 ± 10.67	F	79	80	20	ELISA	7
Yildirim et al.	2016	CSS	Turkey	ACR 1990 & 2010	Age & sex matched healthy controls	No mention	49.4 ± 9.2	50.8 ± 8.8	F	99	99	20	ELISA	6
Maafi et al.	2016	CSS	Iran	ACR 1990 & 2010	Age & sex matched healthy controls	Spring–Autumn (Apr-Sep)	37.96 ± 9.8	32.63 ± 10.1	F	74	68	20	chemiluminescence	6

*CSS* Cross-sectional studies, *RCT* Randomized controlled studies, *ACR* American College of Rheumatology, *FMS* Fibromyalgia syndrome, *NC* Newcastle, *M* Male, *F* Female

The RCT was conducted as a second phase of the study that included both cross-sectional data and an RCT [28, 29]. The two aspects are discussed separately. 10 studies used the ACR 1990 diagnostic criteria to classify the FMS population [12, 18–22, 24–26, 29], while 3 studies used the 2010 criteria in conjunction with the older 1990 criteria [27, 28, 30]. One article did not specify the method of diagnosis [23]. Of the 14 studies, 13 specified the ethnic distribution of included participants, of which 5 were predominantly European populations [12, 21, 23, 24, 28], with the remainder investigating Israeli [18], Egyptian [22], Turkish [19, 25–27], Iranian [30] and Brazilian [20] populations.

### Quality assessment

All thirteen included cross-sectional studies scored between 5 and 7 using the Newcastle-Ottawa score. The most frequent reasons for loss of points on the scale were an apparent lack of comparison between respondents and non-respondents, and a lack of satisfactory or justified sample size. In addition, one study did not specify the method of “ascertainment of exposure” [23], meaning the use of ACR criteria was not mentioned in its specific diagnosis of FMS. One study omitted the tender points examination from diagnosis due to a cited lack of specificity and reproducibility [29].

While the CASP checklist for RCTs is not intended to be used as a tool from which to derive a cumulative score for each study, it was observed that one RCT met 8 of the 10 [28] formative criteria, while the other met 7 [29]. Both RCTs were found to have small sample sizes, increasing the risk of an inaccurately calculated treatment effect and misrepresentation of target population. One of the RCTs also had a 16% dropout rate [29]. The assessments were initially performed by two of the reviewers (SK and JS) and were in high concordance at 95% for cross-sectional studies and 100% for RCTs. Where there was disagreement in quality assessment, both reviewers independently re-assessed the articles until agreement was reached. A third reviewer (SE) reassessed the literature and agreed with the consensus reached by SK and JS.

### Vitamin D levels in fibromyalgia patients and healthy controls

Six studies identified significantly lower vitamin D levels in FMS patients when compared with healthy controls [12, 21, 22, 24, 26, 27]. McBeth et al. investigated men, aged 40–79, in eight European cities in different countries [12]. This large cross-sectional study identified FMS patients to have significantly lower mean vitamin D levels than healthy controls (23.9 ng/ml vs. 25.6 ng/ml; *p* = 0.05) [12]. Furthermore, there were a significantly higher proportion of FMS patients who were classified as having low vitamin D levels (< 15 ng/ml) compared to healthy controls (25.5% vs. 18.6%; *p* = 0.05) [12]. Olama et al., Yildirim et al., Okyay et al. and Al-Allaf et al. also replicated this finding in their studies with smaller cohorts [22, 24, 26, 27]. Interestingly, whilst Atherton et al. also identified a positive relationship between vitamin D deficiency and FMS, they noticed the greatest contrast between FMS patients occurring with vitamin D levels < 30 ng/ml compared with patients who had vitamin D levels between 30 and 40 ng/ml; *p* = 0.001 (OR 1.57, 95% CI 1.09 to 2.26) [21].

A study by Maafi et al. in Iranian women found significantly higher vitamin D levels amongst FMS patients compared to healthy controls (17.2 ng/ml vs. 9.91 ng/ml; *p* = 0.001) [30]. However, the remaining cross-sectional studies found no significant difference in mean vitamin D levels between the FMS patients and healthy controls when no subgroup analysis was applied. The same studies showed no significant difference in the proportion of patients and controls that displayed vitamin D deficiency [18–20, 23, 25, 29]. Tandeter et al. found no significant difference in mean vitamin D levels between FMS patients and controls in pre-menopausal Israeli women (21.75 ng/ml vs. 19.43 ng/ml respectively), and found no significant difference in the proportion of individuals with a vitamin D deficiency between the two groups [18]. Unexpectedly, the proportion of control patients who were vitamin D deficient were found to be slightly higher at 51.2% compared to the FMS patients at 44.1% [18]. However, this was not statistically significant. No differences in vitamin D levels amongst FMS patients and healthy controls were also mirrored in another study conducted in pre-menopausal women [25], and in four other studies conducted on pre- and post-menopausal women [19, 20, 23, 29].

Interestingly, FMS patients have been found to have little seasonal variation in their vitamin D levels compared with healthy controls. A study conducted in Northern Spain by Mateos et al. found a statistically significant increase in vitamin D levels in controls compared to FMS patients after the summer months: 26.9 ng/ml and 23.3 ng/ml, (*p* = 0.03) [23]. However, there was no difference in overall vitamin D levels between FMS patients and controls throughout the year; 23.0 ng/ml vs. 24.0 ng/ml, or in PTH levels; 51.0 vs. 48.0 [23]. The lack of significant difference persisted upon subgroup analysis, finding no distinction between pre- and post-menopausal women for either measurement, although the patient-control difference did become more profound when only considering post-menopausal women (*p* = 0.008) [23].

### Correlation of vitamin D with symptom scores

Unexpectedly, four studies have found an inverse correlation between pain, assessed via the visual analogue score (VAS) or tender points count (TPC), and vitamin D levels [22, 24, 26, 28]; however, the remaining studies could not identify a correlation between the two variables. Several studies have also observed further correlations between vitamin D levels and the presence of other symptoms in FMS patients. The study by Olama et al. found FMS patients with vitamin D levels ≤20 ng/ml to be more likely to have short-term memory impairment, confusion, mood disturbance, sleep disturbance, restless-leg syndrome and palpitations (*p* = 0.05) [22]. They also found inverse correlations with Beck’s depression score; *r* = − 0.328, *p* = 0.020, and lumbar bone mineral density (BMD); *r* = − 0.052, *p* = 0.012 [22]. Interestingly, Wepner et al. also found a significant negative correlation of vitamin D levels with the activities of daily living component of the FMS impact questionnaire (FIQ-ADL); *r* = − 0.344, *p* = 0.030 [28].

### Effect of vitamin D supplementation on pain scores

Warner et al. randomized 50 FMS patients with vitamin D levels between 9 and 20 ng/ml in a double-blind fashion to receive either weekly 50,000 IU vitamin D2 or placebo orally for 3 months [29]. Vitamin D levels were statistically similar at baseline for both groups (*n* = 25) and the vitamin D levels of the treatment group rose significantly higher than that of the placebo group after 3 months; 31.2 ng/ml vs 19.3 ng/ml, *p* = 0.001 [29]. This increase was not met by significant improvements in pain scores in the treated group compared to the placebo group as assessed using VAS, *p* = 0.12, or functional pain score (FPS) [29]. In fact, a significant difference in FPS after 3 months favored the placebo group, *p* = 0.05 [29].

Wepner et al. randomized 30 FMS patients with vitamin D levels < 32 ng/ml in a double-blind fashion to receive either daily 2400 IU (16,800 IU weekly) of vitamin D3 for those with vitamin D levels < 24 ng/ml, or 1200 IU (8400 weekly) for those with levels 24-32 ng/ml, or placebo in FMS patients with vitamin D levels < 32 ng/ml for 25 weeks [28]. One patient was removed from the study as they developed a mild hypercalcaemia (2.71 mmol/L) in response to supplementation. A consistent decrease in VAS score was noted for the treatment group, while remaining stable for the placebo group throughout [28]. A 2 (groups) 4 (time points) variance analysis produced a significant group effect, *p* = 0.025 [28]. No significant difference in vitamin D levels or VAS was noted 24 weeks after stopping supplementation [28]. While no time or group specific effect was noted for the short-form health survey 36 (SF-36), the physical role functioning item of this scale improved significantly from week 1 to week 25 in both supplemented groups, *p* = 0.014 [28]. No significant group-specific effects were observed in depression, anxiety, FIQ-ADL or somatization scores, although the treatment group did experience significantly better outcomes of the FIQ-ADL “morning stiffness” question than the placebo group, at week 13, *p* = 0.007 [28].

## Discussion

This systematic literature review highlights evidence of vitamin D deficiency amongst certain patient populations with FMS; however, there is conflicting evidence regarding supplementation in these patients. There is also large heterogeneity in the measurement of vitamin D across the studies included in this systematic literature review. Assays used to assess 25(OH) levels, which is generally considered to be the best single marker of vitamin D status [31], included enzyme-linked immunosorbent assay (ELISA), radioimmunoassay, chemiluminescent assay and liquid chromatography. This lack of standardization in the measurement of vitamin D makes it difficult to accurately interpret any relationship between serum measurements and clinical deficiency. However, the Vitamin D Standardization Program (VDSP) has attempted to improve the consistency of laboratory measurements of vitamin D and their reporting in clinical studies [32].

The highest quality available evidence indicates significantly lower vitamin D levels in FMS patients compared to healthy controls. The two largest population-based studies by McBeth et al. and Atherton et al. showed evidence of significantly lower mean vitamin D levels in FMS patients and increased odds of deficiency [12, 21], which was also found in the smaller studies by Al-Allaf et al., Olama et al., Yildirim et al. and Okyay et al. [22, 24, 26, 27]. These studies confined their research to homogenous population groups. Indeed, the study by Atherton et al. represented the most robust approach in terms of exhaustively adjusting for known confounders, including BMI, social and lifestyle factors, and the month of vitamin D measurement [21]. Interestingly, a recent meta-analysis by Hsiao et al. involving a large patient cohort of 1854 individuals with chronic pain and 7850 controls found a positive correlation between vitamin D deficiency and chronic pain (crude OR, 1.63; 95% CI, 1.20–2.23), which remained after adjusting for confounders (pooled adjusted OR, 1.41; 95% CI, 1.00–2.00) [33]. Thus, providing strong support for a positive association between hypovitaminosis D and chronic pain conditions such as FMS.

Observational studies have historically implied a link between hypovitaminosis D and conditions associated with chronic pain [3]. However, eight of the studies we analyzed could not draw the same conclusion [18–20, 23, 25, 28, 29], and failed to find an association between vitamin D deficiency and FMS. Of particular note, these studies had smaller patient and control sizes, used more heterogeneous population groups and often did not adjust for important confounders such as BMI, time spent outdoors and clothing [18–20, 23, 28, 29] compared to the studies that found a positive association between lower vitamin D levels and FMS patients. Of particular note, the study by Maafi et al. found an inverse relationship between vitamin D levels in FMS patients compared to healthy controls [30]. The authors speculated that the study participants had easy access to over the counter vitamin D supplements and may have been self-medicating, thus confounding their findings [30].

While a higher prevalence of vitamin D deficiency in FMS patients in six cross-sectional studies has been observed [12, 21, 22, 24, 26, 27], these findings offer little insight into the temporal relationship between disease and deficiency. Indeed, ten of the studies we analyzed were unable to identify a correlation between pain and vitamin D levels [12, 18–21, 23, 25, 27, 29]. Interestingly, preliminary work by Wepner et al. suggested that vitamin D supplementation reduced pain in FMS patients [28]. Warner et al. did not obtain this result or find any beneficial effects to vitamin D supplementation within a larger patient cohort [29]. This is unexpected, as vitamin D is known to modulate proinflammatory cytokine production and central pain processing, thus its deficiency has long been speculated to be involved in chronic pain conditions [34, 35]. In addition, hypovitaminosis D is associated with muscle weakness and pain that improves on supplementation [36]. Both RCTs suffer from limited sample sizes in both treatment and placebo groups [28, 29], which can misrepresent a lack of treatment effect [37]. Thus, highlighting an important need for more RCTs with larger sample sizes to fully establish the role of vitamin D supplementation in treating FMS.

Another important factor to take into consideration is the seasonal and geographical impact on studies investigating the relationship between vitamin D and FMS. Of particular note, the RCT conducted by Warner et al. occurred during the summer months of the year, giving a possible explanation as to why the vitamin D levels in 50% of the placebo group were normalized at the follow up, presumably due to more exposure to sunlight [29]. Several studies have speculated that the physical and mental symptoms accompanying FMS may also dissuade patients from spending time outside in the sun, resulting in a subsequent reduction in their vitamin D levels [22, 30, 38]. Such a pattern has been observed in British Asian rheumatology clinic attendees within the UK [39]. Interestingly, the disparity observed by Olama et al. in the vitamin D levels of Egyptian women provides insight into the broad scale of deficiency among different ethnic groups. Studies suggest that ethnicities with more skin pigmentation are more likely to have vitamin D deficiency [40–42]; however, the variation in vitamin D levels between patients and healthy controls of darker skin tones is difficult to ascertain. This may explain the lack of associations seen in the low powered studies conducted in non-European populations [30, 41, 43].

The studies in this review have not established a clear clinical benefit to vitamin D supplementation in FMS. A recent systematic review by Gaikwad et al. also found no effect by vitamin D supplementation on chronic musculoskeletal pain [44]. Interestingly, the clinical trials by Wepner et al. and Warner et al. used two different forms of vitamin D supplementation (Vitamin D3 and Vitamin D2 respectively). Vitamin D3 is the naturally occurring form of vitamin D, which is also made by skin following UVB light exposure. Vitamin D2 is the derivative of vitamin D3, and commonly found in food. There is currently no clear consensus regarding their efficacy in treating vitamin D deficiency; thus, further studies are needed to identify which of these is the most clinically efficacious and whether vitamin D2 or D3 should be used in future studies regarding the specific physiological benefits of vitamin D supplementation in FMS.

Conversely, as observed by Wepner et al., there is arguably a theoretical risk to supplementation with excessive vitamin D potentially increasing the risk of patient harm through the development of iatrogenic hypercalcaemia [28]. However, this risk is likely minimal. A recent large meta-analysis of vitamin D supplementation in 11,321 participants found that the incidence of adverse events were similar in both treated and placebo groups [45]. In addition, a review of vitamin D supplementation and pain management also concluded that the risks of supplementation in people with deficient levels (defined as 25-hydroxyvitamin levels < 30 nmol/L) are negligible; however, individuals with sufficient levels (25-hydroxyvitamin levels > 50 nmol/L) are unlikely to benefit from additional supplementation [4].

The variation in vitamin D dosages is a particular issue that future studies also need to address. The two RTCs in this review differed in their dosing regimens for supplementation, with Wepner et al. trialing doses of 2400 IU and 1200 IU of vitamin D daily [28] compared with Warner et al. who used 50,000 IU of vitamin D once per week in their RCT [29]. With the European Food Safety Authority suggesting that adults should not exceed 4000 IU (100 micrograms) per day [46], regimens described by Wepner et al. should be sufficient to maintain treatment effect while keeping under the toxic effects threshold of 142 ng/ml [28].

## Conclusion

In summary, the evaluation of the literature suggests a positive association between the diagnoses of FMS and vitamin D deficiency. The evidence is inconsistent, owing to large heterogeneity between studies and the majority of studies possibly being too low powered to display a true effect. Furthermore, treating vitamin D deficiency in FMS has not consistently shown to be of clinical benefit, and excessive supplementation poses a theoretical risk of harm through the development of iatrogenic hypercalcemia. Nevertheless, the limited research into the effect of supplementation on symptom severity in patients with FMS reflects encouraging results that should be repeated in larger studies with a consistent treatment regimen. Future research should focus upon prospective study designs that exhaustively account for confounders, to ascertain any causative nature of vitamin D in the development of FMS. If this tenuous link is developed into a resilient association, vitamin D replenishment represents a cheap, cost-effective method of symptom improvement in patients with FMS. However, for now, the true risk versus benefit of vitamin D supplementation in FMS has not been fully ascertained and should be assessed by clinicians on an individual patient basis.

## Abbreviations

ACR: American College of Rheumatology
CASP: Critical Appraisal Skills Program
FIQ-ADL: FMS impact questionnaire - Activities of daily living
FMS: Fibromyalgia syndrome
PRISMA: Preferred Reporting Items for Systematic Review and Meta-Analyses
RCT: Randomized control trial
TPC: Tender points count
VAS: Visual analogue score
